# Multiplex staining of 2-DE gels for an initial phosphoproteome analysis of germinating seeds and early grown seedlings from a non-orthodox specie: *Quercus ilex* L. subsp. *ballota* [Desf.] Samp.

**DOI:** 10.3389/fpls.2015.00620

**Published:** 2015-08-11

**Authors:** M. Cristina Romero-Rodríguez, Nieves Abril, Rosa Sánchez-Lucas, Jesús V. Jorrín-Novo

**Affiliations:** ^1^Department of Biochemistry and Molecular Biology, University of CordobaCordoba, Spain; ^2^Agricultural and Plant Proteomics Research Group, Department of Biochemistry and Molecular Biology, Escuela Técnica Superior de Ingenieros Agrónomos y de Montes, University of CordobaCordoba, Spain; ^3^Centro Multidisciplinario de Investigaciones Tecnológicas, Universidad Nacional de AsunciónSan Lorenzo, Paraguay

**Keywords:** holm oak, recalcitrant seeds, germination, phosphoproteomics, post translational modification

## Abstract

As a preliminary step in the phosphoproteome analysis of germinating seeds (0 and 24 h after seed imbibition) and early grown seedlings (216 h after seed imbibition) from a non-orthodox sp. *Quercus ilex*, a multiplex (SYPRO-Ruby and Pro-Q DPS) staining of high-resolution 2-DE gels was used. By using this protocol it was possible to detect changes in protein-abundance and/or phosphorylation status. This simple approach could be a good complementary alternative to the enrichment protocols used in the search for phosphoprotein candidates. While 482 spots were visualized with SYPRO-Ruby, 222 were with Pro-Q DPS. Statistically significant differences in spot intensity were observed among samples, these corresponding to 85 SYPRO-Ruby-, 20 Pro-Q-DPS-, and 35 SYPRO-Ruby and Pro-Q-DPS-stained spots. Fifty-five phosphoprotein candidates showing qualitative or quantitative differences between samples were subjected to MALDI-TOF-TOF MS analysis, with 20 of them being identified. Identified proteins belonged to five different functional categories, namely: carbohydrate and amino acid metabolism, defense, protein folding, and oxidation-reduction processes. With the exception of a putative cyclase, the other 19 proteins had at least one orthologous phosphoprotein in *Arabidopsis thaliana, Medicago truncatula, N. tabacum*, and *Glycine max*. Out of the 20 identified, seven showed differences in intensity in Pro-Q-DPS but not in SYPRO-Ruby-stained gels, including enzymes of the glycolysis and amino acid metabolism. This bears out that theory the regulation of these enzymes occurs at the post-translational level by phosphorylation with no changes at the transcriptional or translational level. This is different from the mechanism reported in orthodox seeds, in which concomitant changes in abundance and phosphorylation status have been observed for these enzymes.

## Introduction

Holm oak (*Quercus ilex* L. subsp. *ballota* [Desf.] Samp.) is the dominant tree species in natural forest ecosystems over large areas of the Western Mediterranean Basin (Pulido et al., 2001). Nowadays, forest restoration and reforestation are high priority objectives, with *Q. ilex* being one of the major tree species for such a purpose (MAPA, 2006), this requiring its nursery production at a high scale. *Q. ilex* forest maintenance and sustainability are facing important problems and challenges related to seed viability/conservation and mortality of adult trees and plantlets after field transplantation resulting from adverse environmental conditions like drought and the so-called decline syndrome (Gallego et al., 1999). As natural, non-domesticated, plant species with a great plasticity and phenotypic variability, a key challenge prior to massive clonal propagation is the establishment of techniques for the cataloging and selection of genotypes among provenances with a high survival percentage and productivity under adverse environmental conditions. In our group, a Proteomics Research Program with *Q. ilex* has been carried out in order to study variability of holm oak populations and response to stresses, and to select elite individuals to be used in reforestation programmes (Jorge et al., 2006; Valero Galván et al., 2011, 2012a,b).

Holm oak is a recalcitrant plant species whose germination and viability loss during storage, has been poorly studied at the molecular level if compared with to orthodox ones. This knowledge will help to understand biochemistry and metabolic status before and after the germination process, which could be important for the development and optimization of strategies for large scale propagation, germplasm conservation and seed conservation practices (Balbuena et al., 2011; Walters et al., 2013).

The germination process of plant seeds has been analyzed by using a proteomics approach both for comparative purposes and for characterisation of posttranslational modifications (PTMs), mainly phosphorylation. Phosphorylation is a ubiquitous and reversible PTM, which determines protein conformation, stability and activity (Kersten et al., 2006; Hunt et al., 2007; Bond et al., 2011). Phosphorylation events modulate a wide range of biological processes in plants and other organisms (Nakagami et al., 2010). Thus, in seed germination, phosphorylation has proven to be one of the mechanisms underlying the signaling cascade pathway mediated by ABA (Fujii et al., 2009; Cutler et al., 2010; Umezawa et al., 2013). Quantitative and qualitative profiling of phosphoproteins during seed germination and seedling development has been performed using different proteomic approaches (gel based and gel-free) in different plant species such as *Arabidopsis thaliana* (Sugiyama et al., 2008; Kersten et al., 2009; Reiland et al., 2009), *Medicago truncatula* (Kersten et al., 2009; Rose et al., 2012a), *Phaseolus vulgaris* (Alonso and Zapata, 2014), *Zea mays* (Lu et al., 2008), and *Oryza sativa* (Chen et al., 2014; Han et al., 2014). It is important to highlight that to the best of our knowledge, all previously investigated species produced orthodox seeds and no data on phosphoproteomic analysis of non-orthodox or recalcitrant seeds have been published.

The characterisation of the phosphoproteome includes the detection and identification of phosphoproteins and phosphopeptides, localisation of the exact phosphorylation sites and the quantitation of phosphorylation status, which can be performed by gel-based and gel-free approaches. Although several MS-based approaches for studying phosphoproteins, including down and bottom-up ones (Kaufmann et al., 2001; Woods Ignatoski, 2001; Agrawal and Thelen, 2005) have been used, phosphopeptides are notoriously difficult to analyse, especially in the presence of their non-phosphorylated counterparts. This is due, among other factors, to the low stoichiometry of phosphorylated proteins arising from the fact that only a small fraction of the protein will exist in a particular phosphorylated form (Wu et al., 2011; Rigbolt and Blagoev, 2012).

Phosphoproteomic experiments are being perfomed by using a phospho-protein/peptide enrichment preliminary step (Thingholm et al., 2009). These protocols require an excessive manipulation of the sample, thus reducing the confidence of the comparative results. It is for that reason, and as a complementary protocol, we propose the use of multiplexing (SYPRO-Ruby and Pro-Q DPS) staining of high-resolution 2-DE gels for a simultaneous analysis of protein changes in abundance and/or phosphorylation status.

In the present work we describe the use of that a technique to detect changes in the phosphoprotein profile throughout the *Q. ilex* seed germination and early seedling growth stages. After MALDI-TOF-TOF MS analysis, we have identified 20 proteins whose phosphorylation status varies during the seed developmental process, with seven of them showing no differences in abundance. This last group included enzymes of the glycolytic and amino acid pathways that were, respectively, more and less phosphorylated in seedlings than in seeds. This pattern was different from the one reported for orthodox seed species, in which concomitant changes in abundance and phosphorylation have been observed for enzymes of these two pathways.

## Materials and methods

### Plant material

Mature acorns were harvested during October–November from healthy holm oaks from Cerro Muriano-Córdoba (Córdoba, Spain 37°59′57.74″N, 4°46′57.93″W). Germination and seedling growth were performed at 22 ± 1°C for up to 10 days in darkness as described in Liu et al. (2012). Undamaged, mature acorns were sterilized by immersion in 2.5% sodium hypochlorite, washed abundantly with water and finally dried with filter paper. In order to achive a homogeneous and synchronized germination (Liu et al., 2012), acorns were peeled, removing the pericarp and cutting off parts of the distal ends of the acorns, and then placed in plastic boxes containing one sheet of whatman No3 filter paper over wet perlite. The system was covered with filter paper to avoid water loss (Figure S1). Analyzed time course/periods, corresponding to different seed developmental stages (Figure S2) was selected based on morphology as assessed by microscopic observations (Romero-Rodríguez, 2015; Ph. D Thesis); these stages were selected because they were representative of the morphological changes that occur during germination and seedling growth (24 h after imbibition the emergence of radicle was visible and 216 h shoot seedling started to grow). The embryonic axis was removed from seeds at 0 and 24 h after imbibition and the whole seedling at 216 h after imbibition. Samples from each time were abundantly washed with water, blot dried and frozen in liquid nitrogen and stored at −80°C until protein extraction. Three pooled samples per stage, each one corresponding to a biological replicate (1–2 g fresh weight per pool coming from 20 to 100 individuals), were performed.

### Protein extraction, 2-DE electrophoresis and multiplex staining of the gels

Tissue samples were ground to a fine powder in liquid nitrogen using a mortar and pestle (three biological replicates per stage). Protein extracts were obtained from embryo axes of mature (0 h, un-imbibed seeds) and germinated seeds (24 h after imbibition when the radicle just emerged) and from seedling radicles (4.5–5 cm length, 216 h after imbibition), (Figure S2). Proteins were extracted using TCA/acetone-phenol according to the protocol of Wang et al. (2006). Protein content in samples was estimated by the method of Bradford (Bradford, 1976) with bovine serum albumin as a standard. Samples (400 μg of protein) of each biological replicate per gel, were focused on 17 cm, 5–8 pH IPG strips using a Bio–Rad Protean IEF Cell system (Görg et al., 2004; Maldonado et al., 2008; Valero Galván et al., 2011). The second dimension, SDS-PAGE (Laemmli, 1970) was performed on 12% polyacrylamide gels (PROTEAN® Plus Dodeca Cell). Gels were double stained, first with Pro-Q DPS and then with SYPRO-Ruby (Figure S3) following the procedure described in Agrawal and Thelen (2006) and Berggren et al. (2000). Images were captured with Molecular Imager FX (Bio-Rad Laboratories, Inc.). Experimental *Mr*-values were calculated by mobility comparisons with protein standard markers (SDS-PAGE Standards, 161-0304, Bio-Rad) run in a separate marker lane on the SDS-gel, while pI was determined by using a 5–8 linear scale over the total length of the IPG strips.

### Gel image analysis and statistical tests

Gel image (Pro-Q DPS and SYPRO-Ruby) analysis was performed with PDQuest 8.0.1 software (Bio-Rad) (Valledor and Jorrín, 2011). As reported by Agrawal and Thelen (2006) and in order to eliminate false positives, phosphoproteins spots (revealed with Pro-Q DPS) were only considered if the Pro-Q DPS/SYPRO-Ruby volume ratios were higher than those obtained for negative control, non-phosphorylated markers (β-galactosidase and serum albumin) and with ratios equal to or higher than those obtained for phosphorylated ovoalbumin used as positive control. Consistent spot volumes (those present in all biological replicates) were normalized based on total quantity in valid spots, calculated for each 2-DE gel and used for statistical assessments of differential phosphoprotein and total protein abundance. For statistical analysis (ANOVA, PCA), the web-based software NIA array analysis tool (http://lgsun.grc.nia.nih.gov/anova/index.html) (Sharov et al., 2005; Sghaier-Hammami et al., 2013) was employed.

### MALDI-TOF/TOF analysis

Spots with differential abundance were automatically excised (Investigator ProPic, Genomic Solutions), transferred to multiwell 96 plates, and digested with modified porcine trypsin (sequencing grade; Promega) by using a ProGest (Genomics Solution) digestion station. In-gel digestion was performed as decribed by Shevchenko et al. (1996). Peptides were extracted from gel plugs by adding 10 μL of 10% (v/v) trifluoracetic acid (15 min at room temperature). Solubilized peptides were desalted and concentrated by using μC-18 ZipTip columns (Millipore). Eluate was directly loaded onto the MALDI plate using α-cyano hydroxycinnamic acid as a matrix. Peptide mass analysis was performed with a MALDI-TOF/TOF (4800 Proteomics Analyzer, Applied Biosystems). The most abundant peptide ions were then subjected to fragmentation analysis (MS/MS), providing information that can be used to determine the peptide sequence. Proteins were assigned identification by peptide mass fingerprinting and confirmed by MS/MS analysis. Mascot 2.0 search engine (Matrix Science Ltd., London; http://www.matrixscience.com) was used for protein identification running over non-redundant NCBI protein, UniprotKB, and *Quercus* (Romero-Rodriguez et al., 2014) databases. The following parameters were allowed: taxonomy restrictions to *Viridiplantae* in public databases, one missed cleavage, 100 ppm mass tolerance in MS and 0.5 Da for MS/MS data, cysteine carbamidomethylation as a fixed modification, methionine oxidation, and the phosphorylation of Ser, Thr, and Tyr residues as a variable modification. The confidence in the peptide mass fingerprinting matches (*p* < 0.05) was based on the MOWSE score, and confirmed by the accurate overlapping of the matched peptides with the major peaks of the mass spectrum. Proteins with statistically significant (*p* < 0.05) hits were positively assigned identification. Identified phosphoprotein sequences downloaded from UniprotKB, NCBI nr or available in Quercus_DB (Romero-Rodriguez et al., 2014) were subjected to BLAST analysis by using the phosphoprotein BLAST tool in the Plant Protein Phosphorylation DataBase (P3DB) (Gao et al., 2009) available at http://www.p3db.org/, to find orthologous proteins whose phosphorylation sites were described previously in other species. Proteins identified by MALDI TOF/TOF analysis were extracted and classified based on their putative function according to Kyoto Encyclopedia of Genes and Genomes (KEGG) pathway, using Blast2GO (Conesa et al., 2005) based on BLASTp results against NCBI nr protein database (*e* < 10^−3^), or according to annotations in UniProtKB protein database.

## Results

By using a multiplex double staining of the gels it was possible to detect changes in the protein abundance (SYPRO-Ruby-stained spots) and phosphorylation status (Pro-Q DPS-stained spots) throughout the seed germination and early seedling growth of *Q. ilex* in three different stages, mature seeds (just before imbibition), germinated seeds (24 h after imbibition), and early grown seedlings (216 h after imbibition) (Figure S2). In the analyzed stages the protein yield per fresh weight was around 2–15 mg of protein per g, diminishing during the seedling growth (Table 1).

**Table 1:** **Electrophoretic analysis of changes in the protein and phosphoprotein profile during germination and seedling growth**.

**Hours after imbibition**	**Protein yield mg g^−1^ of fresh weight**	**Spots detected by SYPRO-Ruby stain**	**Spots detected by Pro-Q DPS**	Spots with change in total protein profile^*^ (SYPRO-Ruby)				Spots with change in phosphoprotein profile^*^			
				**Qualitative**		**Quantitative**		**Qualitative**		**Quantitative**	
				**Up**	**Down**	**Up**	**Down**	**Up**	**Down**	**Up**	**Down**
0	15.2	402	205	–	–	–	–	–	–	–	–
24	13.6	412	211	3	3	2	3	1	6	4	2
216	1.9	329	174	17	8	33	23	7	26	12	8

*Up and down accumulated proteins were calculated respect to the mature (0h) stage*.

* *Phosphorylation profile was considered changed when no difference was observed in SYPRO-Ruby staining but was statistically different in Pro-Q DPS. In contrast, it was considered unchanged when a difference was observed in both staining methods*.

*Number of spots detected by SYPRO-Ruby and Pro-Q DPS in different analyzed stages and number of differential spots in total protein and phosphoprotein are shown*.

**Figure 1:**
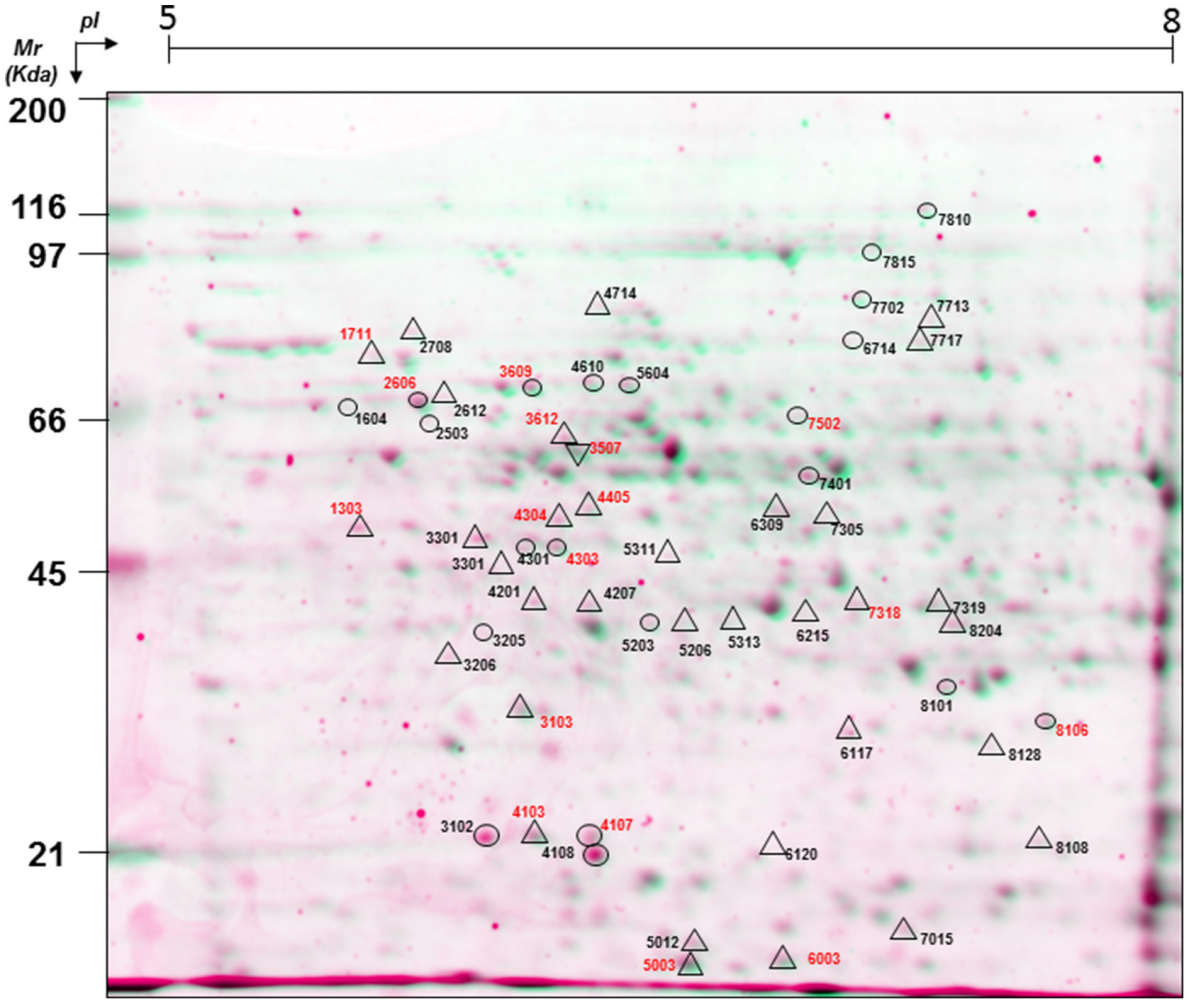
**A virtual 2-DE gel showing the protein profile of ***Q. ilex*** mature seed embryo axis (0 h, un-imbibed) obtained by successive Pro-Q DPS and SYPRO-Ruby staining**. Proteins stained with SYPRO-Ruby appear in green, while Pro-Q DPS stained proteins appear in red. The statistically significant differential phosphoprotein spots are indicated with circles for quantitative differences and with triangles for qualitative (absence/presence) differences. Numbers in red indicate the protein spots that were identified by MALDI TOF/TOF.

Proteins in the extract were separated by 2-DE, and were evenly distributed throughout along the whole pH (5–8) and *Mr* (6–116 kDa) ranges (Figure 1). A total of 482 spots were resolved after SYPRO-Ruby staining, with 222 of them also being stained with Pro-Q DPS, these corresponding to putative phosphoproteins (Table S1).

Consistent Pro-Q DPS stained spots, present in all the three biological replicates, were subjected to statistical, ANOVA and PCA, analysis, with 55, out of the 222, showing significant variations (spot volume) between samples (Table S1). Both qualitative and quantitative changes were observed (Table 1). Taking as a reference the mature seed phosphoprotein-profile, big changes occur after radicle emergence, with small differences in germinated seeds. At the seedling stage (216 h post imbibition), 33 qualitative (7 newly appeared and 26 disappeared), and 20 quantitative (12 up and 8 down) changes were observed (Table 1).

**Figure 2:**
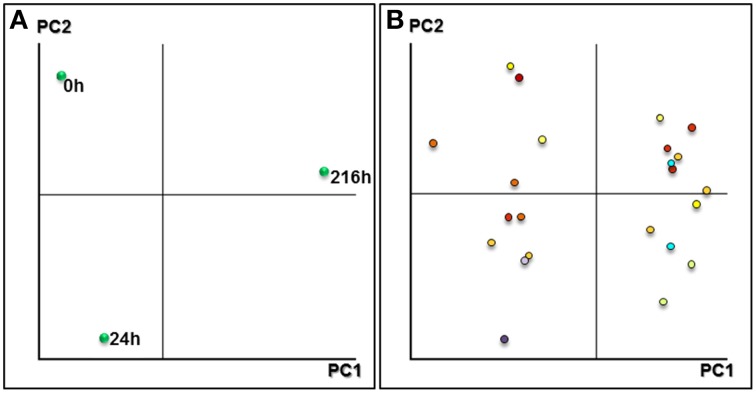
**Principal component analysis plots. (A)** Representation of the samples based on the main principal components found after PCA. **(B)** Plot component PC1 vs. PC2 of differentially expressed spots in three stages analyzed.

Two-dimensional biplots indicating associations between experimental samples and protein spots were generated by principal component analysis (PCA) in NIA array analysis tools (Figure 2). The consistent Pro-Q-DPS stained spots were different enough to establish groups of the samples analyzed. The three analyzed stages were separated from each other; the first component separated the mature (0 h, un-imbibed) and germinated (24 h after imbibition) stage from seedling stages (216 h post imbibition), and the second component separated all the three stages. PCA results showed that PC1 and PC2 explained 88.57 and 11.42% of total variance, respectively. The 55 putative phosphoprotein spots were selected for MALDI-TOF/TOF MS analysis. ANOVA tests of SYPRO-Ruby stained spots (Table S1 and Figure S4) revealed that 20 out of the 55 did not show any differences in abundance while 35 did.

### Protein identification

After MALDI-TOF/TOF analysis, 20 putative phosphoproteins were identified (Table 2). Out of the 20, 13 changed in abundance, while seven did not. For the former the variation in phospho-signal could be simply due to a change in abundance while for the other seven results can only be explained by a modification in their phosphorylation status. To validate the phosphoprotein character/nature of the identified proteins, a BLAST against entries at Plant Protein Phosphorylation DataBase (P3DB; http://www.p3db.org/) (Kersten et al., 2009) was performed. With the only exception of spot 3103, a putative cyclase family protein, the other *Q. ilex* proteins had at least one orthologous phosphoprotein in *A. thaliana, M. truncatula, Nicotiana tabacum*, and *Glycine max*. Table 3 lists the orthologous proteins and their host species (Sugiyama et al., 2008; Jones et al., 2009; Li et al., 2009; Reiland et al., 2009; Grimsrud et al., 2010; Nakagami et al., 2010; Fíla et al., 2012; Rose et al., 2012b).

**Table 2:** **List of proteins identified by MALDI TOF/TOF, grouped in functional categories, based on the KEGG pathways database**.

**Numbers^a^**	**Protein name**	**Accession numbers^b^**	**Mr (pI)**		**MOWSE score^e^**	**Peptide matches**	**Seq cov (%)**	**Fragmented ion (Ion Score)**	**Normalized spot volume^f^**
			**Theor^c^**	**Exp^d^**					
**PHOSPHOPROTEIN SPOTS IN 2-DE WITH CHANGES IN PHOSPHOPROTEIN STATUS**									
**Carbohydrate Metabolism**									
7502	Pyrophosphate-dependent phosphofructokinase beta subunit. *Citrus sinensis* x *Citrus trifoliate*	A9YVC9	62.0 (6.3)	53.8 (6.8)	283	17	22	DKIETPEQFK (59) STGKYYHFVR (45) YYHFVR (37) GQSHFFGYEGR (83)	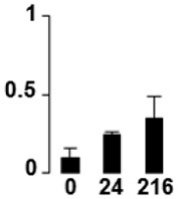
7318	Phosphoglycerate kinase_AT1G79550.1, *Quercus rubra*	QRU405_58	43.5 (6.7)	40.0 (7.1)	231	11	51	YSLKPIVPR (31) VILSTHLGRPK(30) FLKPAVAGFLMQK(21) LVAEIPEGGVLLLENVR(26) LASLADLYVNDAFGTAHR (49)	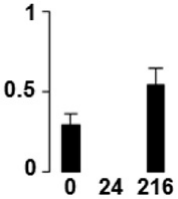
4304	Glucose-1-phosphate adenylyltransferase, *Vitis vinífera*	D7TDB6	56.2 (6.5)	37.4 (6.1)	350	26	38	VDTTILGLDDER (59) KPVPDFSFYDR (71) SSPIYTQPR (41) IINSDNVQEAAR (40)	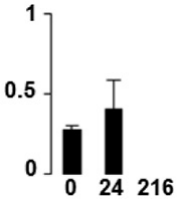
**Amino Acid Metabolism**									
3612	Glutamate decarboxylase_AT2G02010.1, *Quercus* spp.	TC19169_41	58.0 (5.9)	50.2 (6.1)	208	12	45	VVIREDFSR (30) ETPEEIATYWR (53) GSSQIIAQYYQFVR (69) NYVDMDEYPVTTELQNR (46)	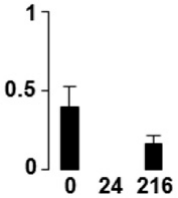
**Protein Folding**									
2606	Heat shock protein 60_AT3G23990.1, *Quercus* spp.	TC33448_39	63.9 (5.6)	68.1 (5.8)	436	11	23	AGIIDPVKVIR (51) IGVQIIQNALK (91) NVVIEQSWGAPK (82) GYISPYFITNQK (73) SDEIAQVGTISANGER (72) AAVEEGIVPGGGVALLYASK (75)	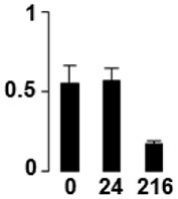
**Unknown**									
4107	Unknown protein	A9PFJ3	29.5 (6.2)	20.2 (6.3)	169	9	35	LQGNYYFQEQLSR (95) GSSIWYGCVLR (29)	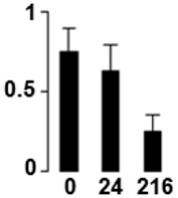
1711	Cell division protein ftsH, putative, *Ricinus communis*	B9S304	75.5 (6.4)	78.7 (5.7)	224	21	26	FLEYLDKDR (48) VRVQLPGLSQELLQK (3) SSGGMGGPGGPGFPLAFGQSK (53) ADILDSALLRPGR (17)	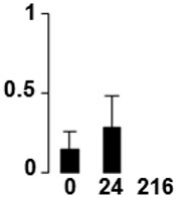
**PHOSPHOPROTEIN WITHOUT CHANGES IN PHOSPHORYLATIONS STATUS**									
**Carbohydrate Metabolism**									
4610	Pyruvate decarboxylase (*Prunus armeniaca*)	B0ZS79	66.2 (5.7)	61.9 (6.2)	235	12	23	ILHHTIGLPDFSQELR(124) EPVPFSLSPR(69)	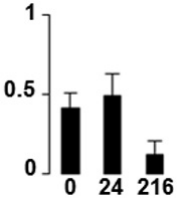
3609	Phosphoglycerate mutase_AT1G09780.1 (*Quercus petraea)*	QP1063_77	61.0 (6.0)	60.5 (6.0)	476	10	25	DAILSGKFDQVR (46) FGHVTFFWNGNR (77) AFEYEDFDKFDR (67) LPSHYLVSPPEIDR (78) GTLHLIGLLSDGGVHSR (98) IQILTSHTCQPVPIAIGGPGLAPGCR (88) AHGSAVGLPTEDDMGNSEVGHNALGAGR (31)	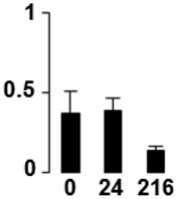
5604	Pyruvate decarboxylase, putative (*Ricinus communis)*	255563082	64.2 (5.9)	61.1 (6.3)	112	2	4	ILHHTIGLPDFSQELR (97) IFVP***S***GVPLK (22) Phosphorilated S379^g^	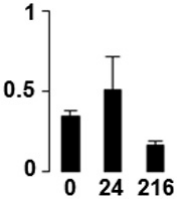
3507	Beta glucosidase 17_AT2G44480.1 (*Quercus* spp.)	QRO15180_40	47.8 (5.2)	60.3 (6.2)	119	10	48	GAYDFIGVNYYTSR (103)	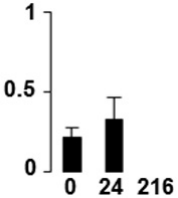
**Amino Acid Metabolism**									
7702	5-methyltetrahydropteroyltriglutamate–homocysteine methyltransferase-like (*Solanum lycopersicum)*	460407874	85.0 (6.0)	82.5 (7.0)	304	4	6	YLFAGVVDGR(76) ALSGAKDEAFF***S***ANAAAQASR(41) Phosphorilated S378^g^EGVKYGAGIGPGVYDIHSPR (64) YGAGIGPGVYDIHSPR(122)	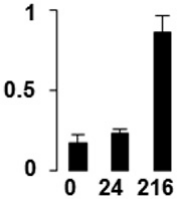
4303	S-adenosylmethionine synthase 2 (*Elaeagnus umbellate*)	Q9AT55	43.6 (5.5)	47.0 (6.2)	650	22	44	TIGFVSDDVGLDADNCK (83) VLVNIEQQSPDIAQGVHGHFTK(97) TQVTVEYYNDKGAMVPVR(17) TIFHLNPSGR(61) FVIGGPHGDAGLTGR(110) FVIGGPHGDAGLTGRK (89) TAAYGHFGR (79)	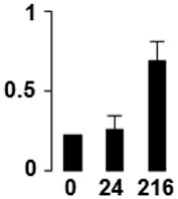
3103	Putative cyclase family protein (*Arachis hypogaea*)	C0L2U1	31.5 (5.04)	30.7 (6.5)	79	8	28	IFDISHR (36)	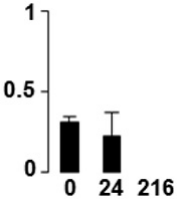
**Oxidation-reduction Process**									
4103	Glutathione S-transferase omega_D6BR66 (*Quercus* spp.)	TC18312_19	28.2 (6.6)	26.0 (6.0)	75	8	36	LYISLSCPYAQR (24) EAGPAFDHLENALSK (14) WIEEVNKIDAYKPTK (11) YIDSNFEGPSLLPNDHAK (24)	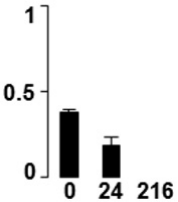
5003	Putative uncharacterized protein (Glutathione-s-transferase theta_B9T0U8) (*Vitis vinífera*)	D7TP00	24.9 (6.2)	15.3(6.6)	107	6	22	NPFGQIPVLEDGDLTLFESR (38) AWWEDISSRPAFK (46)	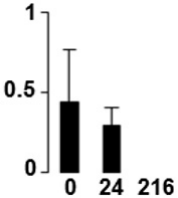
6003	Manganese superoxide dismutase 1_AT3G10920.1 (*Quercus* spp.)	TC29211_11	19.3 (7.9)	15.4 (6.8)	126	5	55	HHQAYITNYNK (73) FNGGGHINHSIFWK (42) KLVVDTTANQDPLVTK(2)	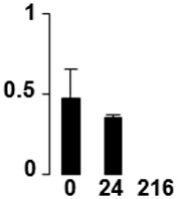
**RNA Metabolism**									
1303	DEAD box RNA helicase (*Pisum sativum*)	Q8H1A5	47.1 (5.4)	49.6 (5.6)	258	24	46	GIYAYGFEKPSAIQQR (60) ILSSGVHVVVGTPGR (28) VFDMLRR (16) MFVLDEADEMLSR (11) VLITTDLLAR (21)	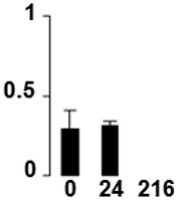
**Stress Response**									
4108	Aluminum induced protein with YGL and LRDR motifs_AT3G22850.1 (*Quercus* spp.)	TC18137_21	27.8 (7.0)	20.7 (6.3)	141	5	27	GCFFTSSGGLR(31) FAFILYDSSSK (49) SYEHPLNEVKPVPR (58) SPEALQSPQSGSVSTLK (4)	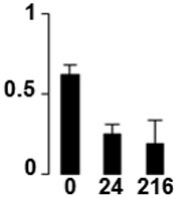
**Unknown**									
8106	AT4G39230.1_ NmrA-like negative transcriptional regulator family protein) (*Quercus robur*)	QRO2324_17	36.5 (6.8)	26.2 (7.6)	367	11	35	AGHPTFALVR(67) VLIIGGTGYIGK(68) FYPSEFGNDVDR(67) AIFNKEDDIGTYTIK(17) NLGVTLVHGDLYDHGSLVK(103 FYPSEFGNDVDRVHAVDPAK(17) GDHTNFEIEPSFGVEASQLYPDVK(33)	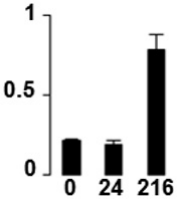

a *Spot number as given on the 2-DE gel images in Figure 1*.

b *Uniprot, NCBI nr and Quercus_DB accessions numbers. The accession whose first letters were TC, QRU, QRO, and QP correspond to Quercus_DB. The accession numbers without letters correspond to NCBI nr*.

c *Molecular weight (KDa) and isoelectric point of each database*.

d *Molecular weight (kDa) and isoelectric point calculated by using molecular weight standards and the PD-Quest Advance (8.01) software*.

e *Mascot score [S = −10 × log(P)]:where P is the probability that the observed match is a random event, peptide matched in MS analysis, percentage of sequence coverage, and ions sequence matched from MS-MS analysis*.

f *The bar charts represent the normalized spot volume vs. analyzed stages*.

g *The phosphorylation sites refer to the position of amino acids in the proteins*.

**Table 3:** **Phosphorylation sites and close species in which the phosphoprotein identified in ***Q. ilex*** germinated seeds and seedling development was identified**.

**Spot number**	**Protein name**	**Protein ID**	**Close species**	**Phosphorylation sites**	**Process in which were described**	**References**
2606	Heat shock protein 60 AT3G23990.1, *Quercus* spp.	TC33448_39	*Arabidopsis thaliana*	474 s	Phosphoproteome characterization of Arabidopsis seedlings shoots and rosette leaves using IMAC and TiO2 phosphopeptide enrichment strategies	Reiland et al., 2009
7318	Phosphoglycerate kinase_AT1G79550.1, *Quercus rubra*	QRU405_58	*Arabidopsis thaliana*	81S 86T 87S		Reiland et al., 2009
1711	Cell division protein ftsH, putative, *Ricinus communis*	B9S304	*Arabidopsis thaliana*	86T		Reiland et al., 2009
1303	DEAD box RNA helicase, *Pisum sativum*	Q8H1A5	*Arabidopsis thaliana*	76S 86S 85Y 105S 716S 723S		Reiland et al., 2009
4107	Unknown protein	A9PFJ3	*Oryza sativa*	235S 261S	Large-scale analysis of rice phosphorylation sites from non-stimulated suspension-cultured rice cells	Nakagami et al., 2010
3612	Glutamate decarboxylase_AT2G02010.1, *Quercus* spp.	TC19169_41	*Arabidopsis thaliana*	8S 10S 13S		Nakagami et al., 2010
4304	Glucose-1-phosphate adenylyltransferase, *Vitis vinifera*	D7TDB6	*Arabidopsis thaliana*	77S		Nakagami et al., 2010
3609	Phosphoglycerate mutase_AT1G09780.1, *Quercus petraea*	QP1063_77	*Arabidopsis thaliana*	82 s	Phosphoproteome role in tobacco pollen activated *in vitro* and large-scale phosphoproteomics	Nakagami et al., 2010; Fíla et al., 2012
3507	Beta glucosidase 17_AT2G44480.1, *Quercus* spp.	QRO15180_40	*Medicago truncatula*	82 T	Large-scale phosphoproteomics analysis in roots	Grimsrud et al., 2010
4610	Pyruvate decarboxylase, *Prunus armeniaca*	B0ZS79	*Nicotiana tabacum*	380T	Phosphoproteome role in tobacco pollen activated *in vitro*	Fíla et al., 2012
5604	Pyruvate decarboxylase, Putative, *Ricinus communis*	gi55563082	*Nicotiana tabacum*	380 T		Fíla et al., 2012
8106	AT4G39230.1_ NmrA-like negative transcriptional regulator family protein, *Quercus robur*	QRO2324_17	*Medicago truncatula*	171 T	Integrated large-scale approach to investigate changes in the phosphoproteome, proteome, and transcriptome that occur 1 h after Nod factors treatment in *Medicago truncatula*	Rose et al., 2012b
4303	S-adenosylmethionine synthase 2, *Elaeagnus umbellate*	Q9AT55	*Medicago truncatula*	131 s 266 s		
4103	Glutathione S-transferase omega_D6BR66, *Quercus* spp.	TC18312_19	*Medicago truncatula*	10 T		
4108	Aluminum induced protein with YGL and LRDR motifs_AT3G22850.1, *Quercus* spp.	TC18137_21	*Arabidopsis thaliana*	215S 216S 240S	Whole cell suspension line, seedlings and seed maturation of rapessed, Arabidopsis and soybean phosphoproteome	Sugiyama et al., 2008; Meyer et al., 2012
6003	Manganese superoxide dismutase 1_AT3G10920.1, *Quercus* spp.	TC29211_11	*Glycine max*	173S	Analysis of seed maturation in Arabidopsis, rapeseed, and Soybean	Meyer et al., 2012
7502	Pyrophosphate-dependent phosphofructokinase beta subunit. *Citrus sinensis* × *Citrus trifoliata*	A9YVC9	*Arabidopsis thaliana*	12T 16S	Nuclear phosphoproteinsanalysis of Arabidopsis	Jones et al., 2009; Reiland et al., 2009
5003	Putative uncharacterized protein (Glutathione-s-transferase theta_B9T0U8),*Vitis vinífera*	D7TP00	*Arabidopsis thaliana*	12 s	Large-scale phosphoproteome analysis of Arabidopsis cell suspension line,	Sugiyama et al., 2008
7702	5-methyltetrahydropteroyltriglutamate homocysteine methyltransferase-like, *Solanum lycopersicum*	460407874	*Arabidopsis thaliana*	698 Y 702 S-		Sugiyama et al., 2008
3103	Putative cyclase family protein, *Arachis hypogaea*	C0L2U1	No hits			

The identified proteins were grouped into functional categories based on the KEGG pathways database (Table 2): carbohydrate and amino acid metabolism, defense, protein folding and oxidation-reduction processes.

## Discussion

As a preliminary step in the phosphoproteome analysis during the seed germination and early seedling growth processes of a non-orthodox sp. *Q. ilex*, a multiplex (SYPRO-Ruby and Pro-Q DPS) staining of high-resolution 2-DE gels was used. With this protocol it was possible to detect changes in protein-abundance and/or phosphorylation status, identifying, at the same time, candidate phosphoproteins. This simple technique could be a good complementary alternative to the enrichment protocols used in the search for phosphoprotein (Subba et al., 2013; Han et al., 2014; Li et al., 2015). Phosphoprotein enrichment apart from providing, as Pro-Q staining does, false positives, involves excessive manipulation of the sample that results in protein and PTM losses and possible biases. It is true that phosphoprotein validation requires the identification of the phosphorylated peptide, this not being possible or being moret difficult through the MALDI-TOF-TOF MS strategy employed in this work (Thingholm et al., 2009). The protocol presented suffers from the inherent limitations of the 2-DE coupled to the MALDI-TOF-TOF strategy, such as the possible existence and identification of commigrating spots. In at least one case, that of the cyclase, a phosphopeptide was identified, thus confirming its phosphoprotein nature. In any case, rather than identifying the site of phosphorylation, our objective was to search for putative phosphoroteins that showed changes in the phosphorylation status and interpreted those changes from a biological point of view. Different evidence confirmed that most of the Pro-Q–DPS stained spots identified corresponded to real phosphoproteins. Thus: (i) they are Pro-Q stained; (ii) they were only considered if the Pro-Q DPS/SYPRO-Ruby volume ratios were higher than those obtained for a negative control, non-phosphorylated markers (β-galactosidase and serum albumin in this work) and with a ratio equal to or higher than to those obtained for phosphorylated ovoalbumin used as a positive control (Agrawal and Thelen, 2006); (iii) phosphoprotein orthologs have been reported for four different plant species, including *A. thaliana, M. truncatula, N. tabacum*, and *G. max;* (iv) biological interpretation of the data, as discussed below, fits in very well with what is known about the regulation of the identified proteins by phosphorylation. The percentage of phosphorylated proteins detected in our experimental system was of 46%, with similar figures reported for chickpea seedlings (300, Subba et al., 2013) but lower than those reported for germinating rice seeds (500, Han et al., 2014). This could be due to the different methodological approaches and the experimental system used, rather than the system itself or the biological process used or the experimental conditions rather than differences in the number of detectable phosphorylated proteins.

The percentage of protein identification was lower than that obtained in Coomassie stained spots (Valero Galván et al., 2011, 2012b), this being related to the amount of protein present beyond the absence of sequences for *Quercus* in databases. The most important functional categories are discussed. In this work, we paid special attention to those proteins that showed variations in the phosphorylation pattern with no changes in protein abundance, so that they were supposed to be regulated at the post-translational levels. Independent (protein abundance or phosphorylation pattern) or simultaneous, multiplex (protein abundance and phosphorylation pattern) proteomics analysis by using a similar (bottom-up, 2-DE based) strategy has been used in the analysis of mature orthodox seed and seed germination process in model and crop plant species, including *Arabidopsis*, rice, soybean, rapeseed and maize (Lu et al., 2008; Meyer et al., 2012; Han et al., 2014).

**Figure 3:**
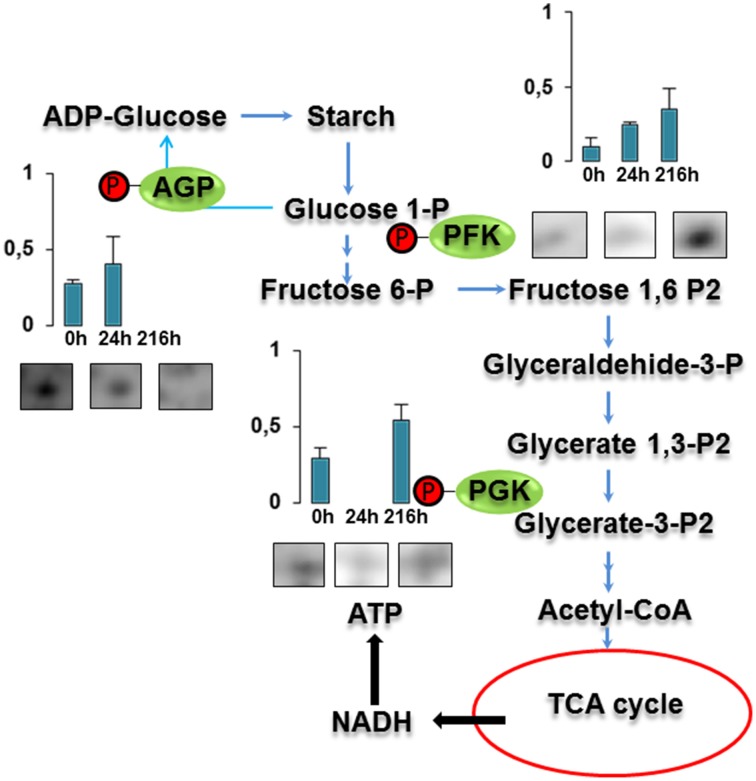
**Carbohydrate metabolic pathway constructed with proteins whose phosphorylation changed**. Proteins are represented by a green circle. The figures represent the normalized spots volume vs. analyzed stages of each protein, also shown is the spot volume in the stages analyzed.

Three proteins belonged to the *carbohydrate metabolism* category: pyrophosphate-dependent phosphofructokinase (PPi-PFK, spot 7502), phosphoglycerate kinase (PGK, spot 7318) and glucose-1-phosphate adenylyltransferase (AGP, spot 4304) (Table 2 and Figure 3). PPi-PFK is a cytosolic enzyme that catalyzes the phosphorylation of fructose-6-phosphate to fructose-1,6-bisphosphate in the glycolytic direction, using inorganic pyrophosphate as the phosphoryl donor. This process makes fructose flow into glycolysis to provide energy. PGK catalyzes the conversion of 1,3-diphosphoglycerate to 3-phosphoglycerate, the first substrate-level phosphorylation reaction in the glycolytic pathway for production of ATP. AGP catalyzes the synthesis of ADP-glucose, which is the active glucoside for starch synthesis (Figure 3). Overall, the phosphorylation states of these three enzymes increased throughout the germination process. Phosphorylation modification of many glycolytic enzymes has been reported to cause a significant increase in enzyme activity (Li et al., 2011), incrementing the glycolysis rate and the generation of energy to supply the needs of the developing seedling. These results are in agreement with previous studies on rice germination and seedling (Nakagami et al., 2010; Chen et al., 2014; Han et al., 2014). An increased glycolytic activity in germinating *Q. ilex* seeds is supported by a decrease in sucrose content (Romero-Rodríguez, 2015).

On the contrary, for enzymes of the amino acid metabolism (Glutamate decarboxylase, spot 3612) and chaperones (Heat shock protein 60, spot 2606) a decrease in their phosphorylation signal was observed (Table 2). Glutamate decarboxylase (GDC) catalyzes the decarboxylation of glutamate to GABA, a non-protein amino acid involved in stress tolerances that accumulates in germinating seeds of rice and tomato (Taji et al., 2002; Leitner et al., 2011). Some isoforms of this enzyme are inhibited by phosphorylation (Bao et al., 1995). If applicable to GDC, the reduction in its phosphorylation status observed here might imply an increase in the activity of this enzyme, to eliminate the excess of glutamate and glutamine originated by the high rates of stored proteins degradation occurring during germination.

The phosphorylation status of heat shock proteins (HSPs), involved in *protein folding*, has been described decreasing during rice germination (Han et al., 2014). In agreement with that, HSP60 showed high levels of phosphorylation in non-imbibed (0 h after imbibition) and germinated seeds (24 h after imbibition).

In conclusion, over 200 putative phosphoproteins spots were detected in our analysis. Among them, 20 proteins exhibited significant changes in their phosphorylation status, seven of which were identified. Identified enzymes of the glycolytic (pyrophosphate-dependent phosphofructokinase and phosphoglycerate kinase) and amino acid metabolic pathways (glutamate decarboxylase) and protein folding (heat shock protein 60) did not change in abundance during germination and growth but their phosphorylation status increased suggesting regulation at the post-translational level. Alterations in the phosphorylation status of proteins related to glycolysis and amino acid metabolism are in agreement considering that these pathways must increase from mature seeds to germinated seeds and seedling. To test these hypotheses it is necessary identify the phosphorylation sites, but this work constitutes an initiation in the study of the molecular mechanism involved in *Q. ilex* seed germination. The phosphoproteome analysis suggested that the metabolic machinery present in the recalcitrant seeds receives a signal to activate and resume/summarize the most important metabolic pathways in *Q. ilex* to start the germination and the establishment of the seedlings. In orthodox seeds, changes in abundance, together with differences in their phosphorylation status, were observed for these enzymes. Thus, this is one of the differences between orthodox and non-orthodox seeds that may explain their different behavior (Han et al., 2014).

### Conflict of interest statement

The authors declare that the research was conducted in the absence of any commercial or financial relationships that could be construed as a potential conflict of interest.
