# Supplementary material for: Multiplex staining of 2-DE gels for an initial phosphoproteome analysis of germinating seeds and early grown seedlings from a non-orthodox specie: Quercus ilex L. subsp. ballota [Desf.] Samp
Source: Front Plant Sci. 2015 Aug 11;6:620. doi: 10.3389/fpls.2015.00620 (PMC4531236; doi:10.3389/fpls.2015.00620)
Supplement: Supplementary file 2 [file Presentation1.PDF]

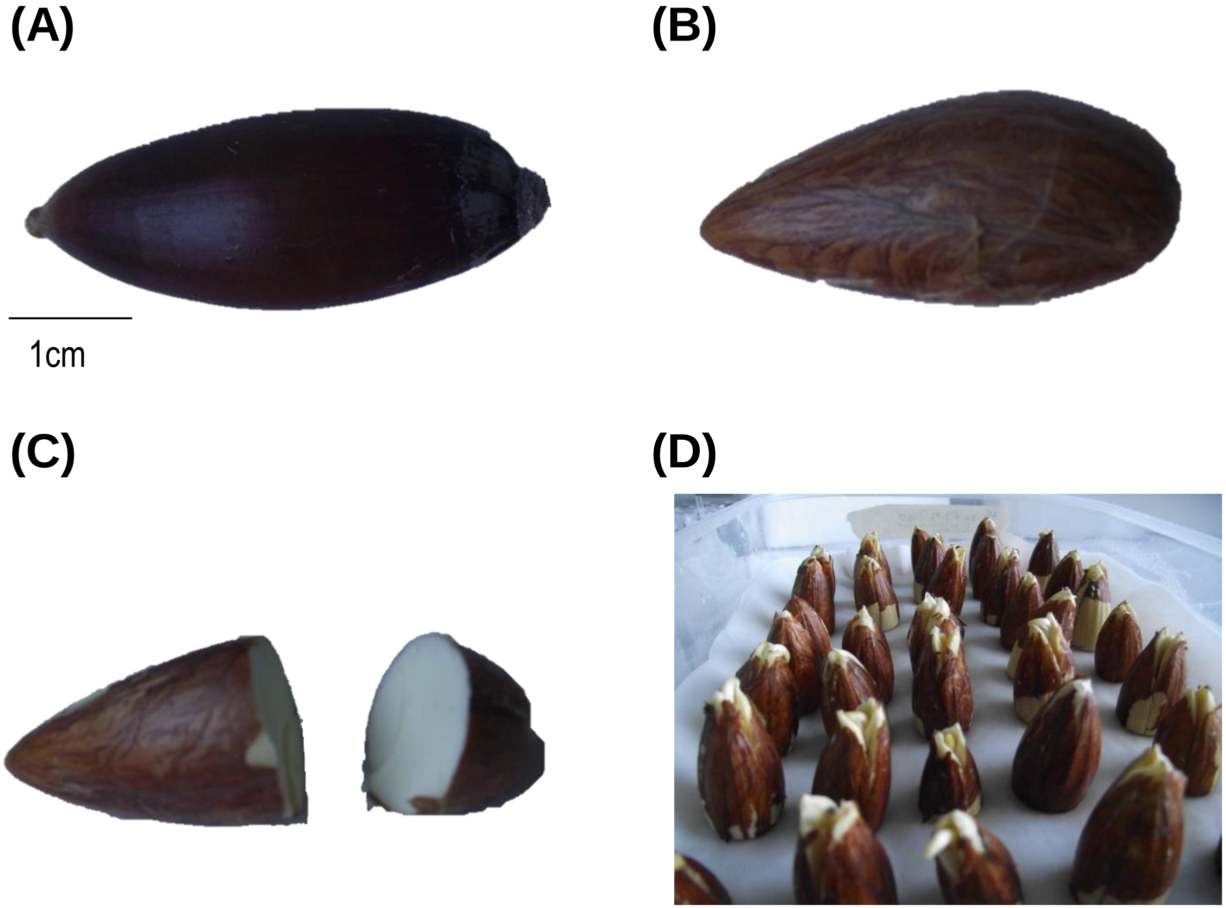

Figure S1: Process in acorn germination. (A). Acorn with pericarp **(B).** Dehulled acorn **(C).** Cut acorn in the distal **(D).** Boxes with filter paper and perlite with germinated acorn.

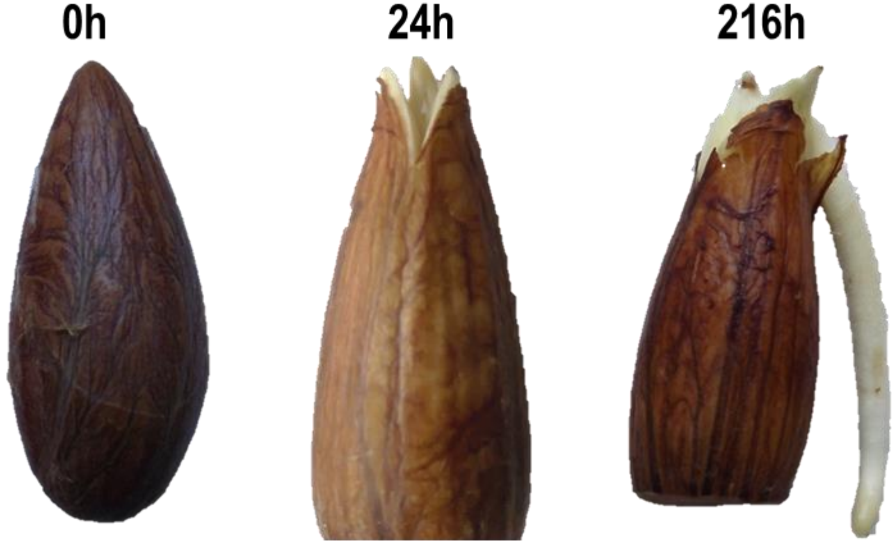

Figure S2: Morphological aspect of the acorns and seeds at the sampling stages. The time approximate to obtain the indicated stage, in hour post imbibition is indicated.

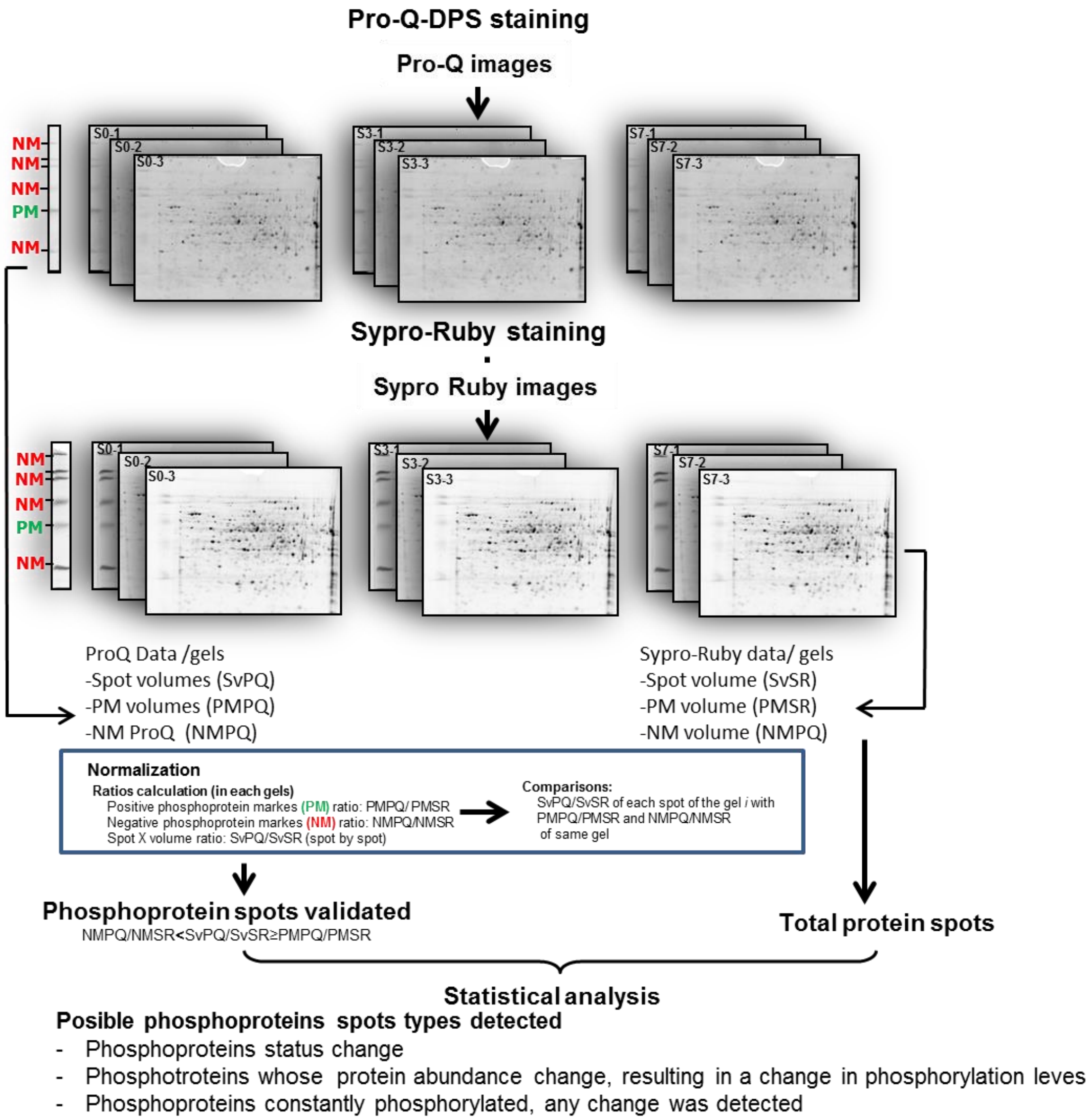

Figure S3: Phosphoproteome analysis workflow by multiplex-staining of high-resolution 2-DE gels. Pro-Q DPS was used to reveal phosphoproteins and SYPRO-Ruby for total proteins.

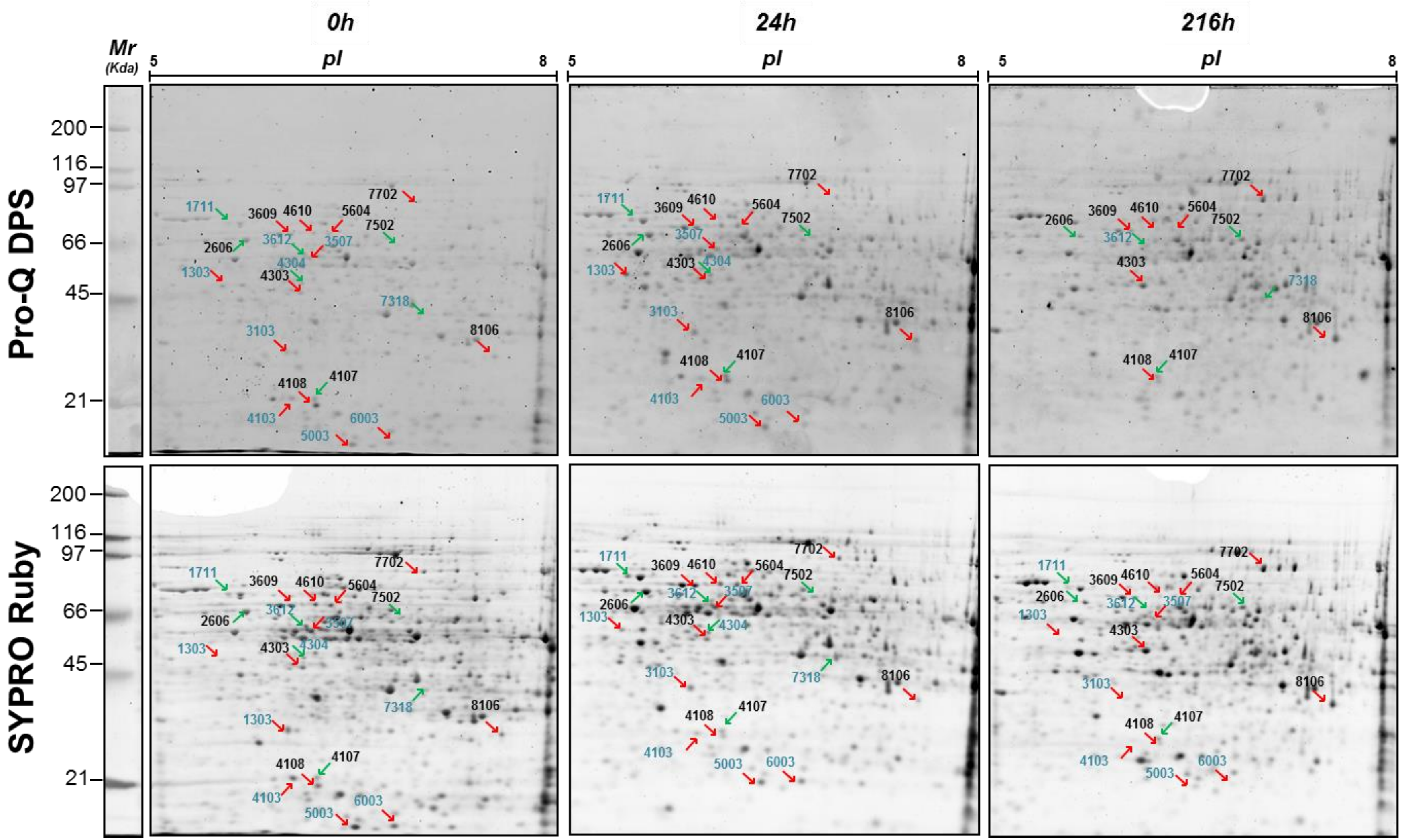

Figure S4. Representative 2-DE phosphoproteome profiles of *Q. ilex* embryos during germination and seedling. The gels were successively stained with Pro-Q DPS and SYPRO-Ruby as indicated in the figure and the images captured and analysed. Arrows indicate the identified proteins and the colors indicate the changes in phosphorylation status (green) or in protein levels (red).
